# Genomic analysis of bacteriophage ε^34 ^of *Salmonella enterica *serovar Anatum (15+)

**DOI:** 10.1186/1471-2180-8-227

**Published:** 2008-12-17

**Authors:** Robert Villafane, Milka Zayas, Eddie B Gilcrease, Andrew M Kropinski, Sherwood R Casjens

**Affiliations:** 1 Ponce School of Medicine, Department of Microbiology, Ponce, Puerto Rico 00732; 2 Ponce School of Medicine, Department of Biochemistry, Ponce, Puerto Rico 00732; 3 Department of Pathology, 5200 Emma Eccles Jones Research Building, U. of Utah School of Medicine, 15 North Medical Drive East, Salt Lake City, UT 84112, USA; 4 Department of Microbiology and Immunology, Queens University, Kingston, Ontario, Canada, K7L 3N6; 5 Public Health Agency of Canada, Laboratory for Foodborne Zoonoses, Guelph, Ontario, Canada, N1G 3W4; 6 Current address : Alabama State University, Program in Microbiology, Department of Biological Sciences, 915 S. Jackson Street, Montgomery, AL 36101

## Abstract

**Background:**

The presence of prophages has been an important variable in genetic exchange and divergence in most bacteria. This study reports the determination of the genomic sequence of *Salmonella *phage ε^34^, a temperate bacteriophage that was important in the early study of prophages that modify their hosts' cell surface and is of a type (P22-like) that is common in *Salmonella *genomes.

**Results:**

The sequence shows that ε^34 ^is a mosaically related member of the P22 branch of the lambdoid phages. Its sequence is compared with the known P22-like phages and several related but previously unanalyzed prophage sequences in reported bacterial genome sequences.

**Conclusion:**

These comparisons indicate that there has been little if any genetic exchange within the procapsid assembly gene cluster with P22-like *E. coli/Shigella *phages that are have orthologous but divergent genes in this region. Presumably this observation reflects the fact that virion assembly proteins interact intimately and divergent proteins can no longer interact. On the other hand, non-assembly genes in the "ant moron" appear to be in a state of rapid flux, and regulatory genes outside the assembly gene cluster have clearly enjoyed numerous and recent horizontal exchanges with phages outside the P22-like group. The present analysis also shows that ε^34 ^harbors a *gtrABC *gene cluster which should encode the enzymatic machinery to chemically modify the host O antigen polysaccharide, thus explaining its ability to alter its host's serotype. A comprehensive comparative analysis of the known phage *gtrABC *gene clusters shows that they are highly mobile, having been exchanged even between phage types, and that most "bacterial" *gtrABC *genes lie in prophages that vary from being largely intact to highly degraded. Clearly, temperate phages are very major contributors to the O-antigen serotype of their *Salmonella *hosts.

## Background

Recent studies of tailed-phages have shown their enormous numbers and wide overall diversity [1-3], but relatively few studies have had as a long-range goal an attempt to analyze the range of diversity in tailed-phages that infect a particular bacterial species or cluster of species (see, for example, [4,5] and references therein for the phages of *Mycobacterium*). We are interested in the diversity of dsDNA tailed-bacteriophages that infect *Salmonella*; such analyses of enteric bacteria enjoy the major benefit of the huge amount of previous genetic and biochemical characterization of the proteins encoded by the *Escherichia coli *and *Salmonella enterica *"model system" phages. Many members of the *Caudovirales *(tailed-phages) specific for *Salmonella *species have been reported, but no careful comparison of these many phages has been done with a goal of understanding the detailed nature of the diversity of these viruses. In 1950, Boyd recognized that *S. enterica *serovar Typhimurium carried "symbiotic bacteriophages", which we now call prophages [6]. Genomic sequencing has shown that essentially all Salmonellae carry prophages [7-13]. Some of these prophages are fully functional and some are clearly defective and no longer competent to program a complete phage lytic cycle.

Temperate phages often modify the host cell surface lipopolysaccharide upon lysogenization in a process called "lysogenic conversion." *Salmonella *phage ε^34^, the subject of this report, is a temperate phage that was isolated in the 1950s [14] that was historically important in the proof of this phenomenon [14-20]. Previous studies have provided an early genetic map for ε^34^, and identified the conversion genes as well as the virion O-antigen polysaccharide receptor-recognizing tailspike encoding gene [19-25], and ε^34 ^will only adsorb to and thus infect its *Salmonella enterica *serovar Anatum host cell if the latter carries an ε^15 ^prophage [15,18]. This type of modification of surface polysaccharides has been implicated in bacterial virulence [26]. We report here the genome sequence of phage ε^34 ^and compare it to its relatively close P22-like relatives.

## Results and discussion

### 1. Analysis of the ε^34 ^genome sequence

Double-stranded DNA from ε^34 ^carrying the clear plaque mutation(s) c99 was sequenced. The alterations in the clear mutant c99 strain were determined to affect genes *46 *and *48 *(see below). The sequencing runs assembled into a single circular sequence, and its annotated genome sequence is arbitrarily opened at the 5'-end of the small terminase subunit gene according to convention with this type of phage. The genome is 43016 bp long and is 47.26% G+C which is somewhat lower than the host bacterium 53% [27]. The ε^34 ^genome is predicted to contain 71 protein-coding genes (shown diagrammatically in figure S1 in the Additional file 1) and genes for at least two antisense RNAs, Sar and Q antisense RNA. A complete list and description of the genes is presented in Table S1 as Additional file 1. Comparison of proteins predicted to be encoded by these 71 genes with the extant database (BLASTP and PSI-BLAST [28]) along with the gene order and orientation show clearly that ε^34 ^is a P22-like member of the larger "lambdoid" phage group. Its morphogenetic genes show a clear one-to-one relationship with those of phage P22, while its early regions show a typical mosaic relationship with other lambdoid phages. Of the 71 predicted genes, only seven (genes *15, 40, 44, 45, 59, 60*, and *63*) do not have a phage- or prophage-borne match in the current database; of these, gene *15 *matches numerous bacterial genes of unknown function. Space limitations preclude citation of all the molecular and genetic studies that have lead to the current understanding of the genes in the lambdoid phages (see refs. [29,30]).

### 2. The ε^34 ^genes

#### a. Early left operon

The ε^34 ^early left operon contains 21 open reading frames, all of which have homology to genes in currently known lambdoid phages. The first gene in this operon, gene *43*, encodes a homolog of the highly studied λ transcriptional antitermination N protein that has high similarity in its C-terminal region (AAs 28–120) to the C-terminal regions of orthologues in other lambdoid phages, however its N-terminal 27 AAs are at best only 62% identical to those proteins. Since the C-terminal part is thought to be involved in association with host RNA polymerase and the BoxB-nut RNA site [29,30], it seems likely that the ε^34 ^protein has a novel BoxB specificity. Immediately transcriptionally downstream (left) of gene *43*, genes *31 *through *42 *are somewhat similar to and syntenic with the parallel region of P22-like phage ST64T. These include homologues of phage λ *cIII *(gene *39*) and *kil *(gene *38*) genes, which control establishment of lysogeny and inhibition of host septation, respectively. Genes *33 *through *36 *encode the following proteins that are likely to be involved in catalyzing homologous recombination: a P22 type anti-RecBCD protein Abc2, a possible endonuclease, a bacterial type single-strand DNA binding protein, and a P22-type Erf ("essential recombination function") protein. The putative gene *34*-encoded nuclease is unusual in this context, and gene *36 *is called a homologue of the well studied phage P22 Erf protein only because their C-terminal 56 amino acids are 86% identical. Parallel Erf "domain swaps" have been discussed previously [31-33], and these are consistent with the known structure of the protein [34]. Downstream gene, *31*, appears to be an in-frame fusion of the N-terminus of a gene that is similar to *eaE *found in this location in the P22 genome and a putative HNH homing endonuclease. Interestingly, the latter is homologous to endonucleases from phage infecting Gram-positive bacteria including *Lactobacillus *phages A2 (NP_680539.1) and ΦAT3 (YP_025078.1). Downstream genes *29 *and *30 *encode proteins that are identical to P22 EaF and Orf45, respectively, while genes *25*-*28 *specify proteins that are closely related to those of ε^15^ genes *48 *through *45*, respectively (*e.g*., ε^34 ^gp28 and ε^15^ gp45 are 96% identical over their 277 amino acids). Finally, genes *23 *and *24 *are oriented like their P22 homologues and encode putative integrase and excisionase proteins that are discussed in more detail below.

#### b. Early right operon

The ε^34 ^early right operon contains sixteen genes, all but two of which are homologous to known lambdoid phage early right operon genes. The first two genes in this operon are similar to lambdoid *cro *and *cII *genes, respectively, and the next four genes, *49 *through *53*, are very similar to the phage ES18 DNA replication gene region; of these, ε^34 ^gp50 is likely the origin binding protein because of its λ gpO homology and gp51 is a putative helicase [33]. Genes *54 *through *61*, represent a typically mosaic lambdoid "nin region" that includes homologues of λ and P22 *ninA, B, D, E, F *and *Z *genes. Only the short gene *59 *has no known homologues and gene *60 *only has two closely related (but unannotated) homologues in *Sodalis glossinidius *prophages [35]. The last gene in the operon (*62*) encodes a protein that is 98% identical to the phage λ gene *Q *antitermination protein, and the putative Q protein target (*qut*) in ε^34 ^is identical to that of phage λ, indicating that their target specificities are the same.

#### c. Late operon

The lambdoid phage late operons are turned on by Q antitermination and encode the genes for virion assembly and cell lysis. The ε^34 ^virion is indistinguishable from the P22 virion by negative stain electron microscopy [21], and most of the morphogenetic genes of ε^34 ^are closely related to those of P22. The only major differences are in ε^34 ^genes *6, 12, 13 *and *19*. The P22 gene *6 *homologue is partly deleted relative to ε^34 ^(see below). Homologues of genes *12 *and *13 *encode proteins in P22 that are released with the DNA during injection, and *19 *encodes the tailspike (discussion of the latter below). SDS polyacrylamide electrophoresis gels of the proteins of the ε^34 ^virions are nearly identical to those of P22 except for a small size difference in the ejection protein gp13, the tailspike protein and, the presence of a strong ~15 kDa band that is not present in P22 virions [21,22]. N-terminal sequence analysis of the latter band excised from such gels gave a sequence of NH_2_-ANPNF (performed as described in [36]), indicating that it is encoded by gene *71*, and that its N-terminal methionine is removed. This protein is 97% identical to the Dec protein of phage L that has been shown to make virions more stable to magnesium ion chelation [36].

#### d. Integration

By homology with other lambdoid phages that integrate into tRNA genes, the putative integration attachment site (*attP*) of ε^34 ^is found just downstream of the *int *(*23*) gene within a 43 bp sequence that is identical to part of the tRNA *argU *gene. This strongly suggests that ε^34 ^integrates into, and in the process replaces, the 3'-portion of the *argU *gene in the *Salmonella *chromosome. The closest known relative to ε^34 ^integrase is that of the *E. coli *K12 defective prophage DLP12 which is also integrated into *argU *[37]. Although these two integrases are only about 80% identical in sequence, most of the differences are outside the following very similar regions: amino acids 1–80, 145–260 and 300–360. These similar regions include or overlap the target specificity generating "arm binding" N-terminal (AAs ~1–60) and "core binding" (AAs ~75–175) domains of λ integrase [38], so identical target specificity of the ε^34 ^and DLP12 integrases is reasonable. We note that several *Salmonella *P22-like phages that integrate into the tRNA *thrW *gene, P22, ST104 and ST64T, have integrases that are about 30% different from ε^34^.

#### e. Lysogenic conversion

**Figure 1:**
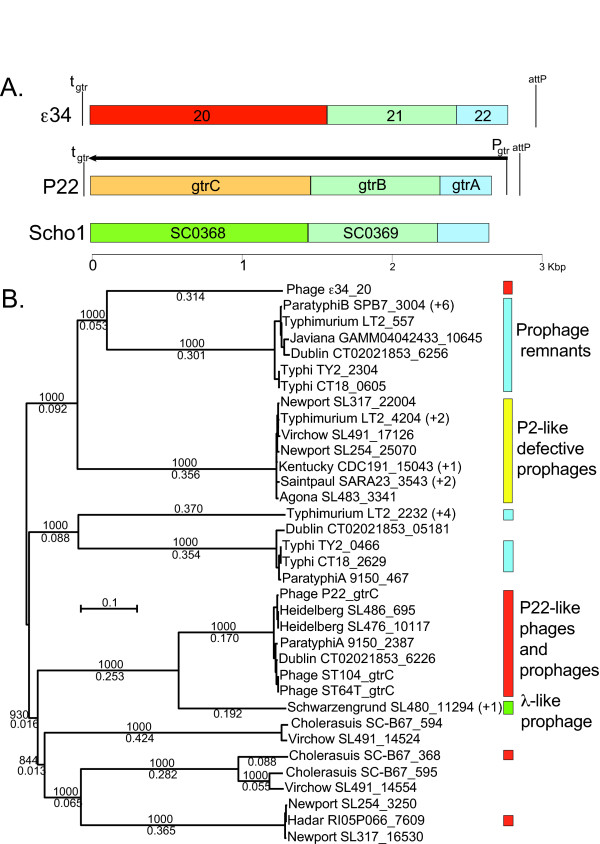
**O-antigen modifying operons in *Salmonella *bacterial and phage genome sequences**. A. Examples of *GtrABC *operons. Operons are shown for phage ε^34 ^and P22, as well as for a possibly intact P22-like prophage, which we call Scho1, in the genome of *Salmonella enterica *serovar Choleraesuis strain SC-B67 (where the putative *gtrC *gene is named SC0368); genes are indicated as colored rectangles, and rectangles of the same color are recognizably homologous to one another. The transcription of the P22 operon is indicated by a horizontal arrow. B. A CLUSTAL X2 [80] generated neighbor-joining tree of fifty-one known *Salmonella *GtrC proteins. Horizontal distances in the tree are proportional to sequence differences, and bootstrap values (out of 1000 trials) are shown above the lines and sequence differences are shown below the lines for the long branches. Each GtrC protein is named on the right of the tree according to its location with its "phage" or "*Salmonella *serovar" name followed by and underlined space and the numeric portion of its GenBank "locus_tag"; "+ number" in parentheses indicates the number of additional identical sequences currently known in other *Salmonella *strains. On the far right, colored bars indicate the type of prophage that is associated with the *gtrC *gene; red, P22-like; yellow, P2-like; green, λ-like; and blue, prophage remnant of uncertain ancestry.

Phage ε^34 ^has at least six genes (*16, 20, 21, 22, 44*, and *45*) that should be, by their position and experimental work on homologues in other phages, expressed from the prophage. Two of these, *44 *and *45*, appear to be in the same operon and downstream of the prophage repressor gene (*46*) gene (like *rexA *and *rexB *in λ), but they have no matches in the current sequence database. Genes *16 *is a homologue of the P22 *mnt *maintenance repressor gene discussed below in the context of the "ant moron". Genes *21 *and *22 *have similarity to genes known to be involved in surface polysaccharide modification, and ε^34 ^is known to perform such modifications [18,19,39]. Genes *22 *and *21 *proteins are very similar to phage-encoded bactoprenol-linked glucose flippases that translocate glucosyated undecaprenyl phosphate from the cytoplasmic face to the periplasmic face of the inner membrane and bactoprenol glucosyl transferases that catalyze transfer of the glucose from UDP-glucose to the above prenyl intermediate, respectively. Genes in these two families are designated *gtrA *and *gtrB *[26,40], respectively. In addition, highly sequence variable but similarly sized genes encoding integral membrane proteins, called *gtrC *here, are found immediately transcriptionally downstream of *gtrA *and *gtrB *(in the position of ε^34 ^gene *20*) in similar operons in numerous other phages, prophages, and bacterial chromosomes (see below). In the best studied cases, *Shigella flexneri *temperate phages SfII, SfV and SfX, their similar operons are known to be responsible for enzymatic modification of the O-antigen polysaccharide to give "serotype conversion" of the bacterial host by the prophage. These downstream variable genes have in some cases been shown to be serotype-specific glucosyl transferases that add various glucosyl residues to the O-antigen backbone repeat. The first two genes, *gtrA *and *gtrB*, are highly conserved and not thought to be serotype specific, while *gtrC *genes are serotype specific [26]. Thus, the ε^34 ^genes *20–22*, which appear to constitute an operon since the three genes are arranged with very little or no space between them, are almost certainly responsible for the conversion of *Salmonella *from serotype 3,10,15 to serotype 3,15,34 by the ε^34 ^prophage (figure 1A) [14-20].

The ε^34 ^gene *20 *protein is moderately similar (30–40% identity) to proteins encoded by genes that lie in the *gtrC *position of several otherwise very similar operons in *S. enterica *genomes (*e.g.*, Typhimurium LT2 (locus_tag STM0557) and serovar Typhi CT18 (locus_tag STY0605). Further analysis of the currently available (March 2008) database results in fifty-one "*gtrC*" genes, most of which lie in apparently intact operons with adjacent *gtrA *and *gtrB *genes in bacterial genomes. These putative *gtrC *genes fall into nine different sequence types whose encoded proteins are at best 40% identical (figure 1B). Some of these types have very little sequence similarity (*e.g*., ε^34 ^gene *20 *protein and phage ST64T GtrC). GtrC type proteins GtrII, GtrV and GtrX, encoded by *Shigella *phages (above), have been shown to have nine transmembrane helices [41-43], and all of the nine types of GtrC protein in the *Salmonella *operons are very strongly predicted to have between nine and eleven transmembrane helices (by TopPred II analysis [44]). The ε^34 ^GtrC protein, which is predicted to have nine or ten transmembrane segments, is a unique type. We note that a substantial majority of the 47 "bacterial" *gtrC *genes in figure 1B are adjacent to *gtrB *and *gtrA *genes and reside in prophages. These include P22-like prophages in the genomes of *Salmonella *serovars Arizonae, Choleraesuis, Dublin, Hadar, Heidelberg, and Paratyphi A; and; apparently defective phage P2-like prophages in serovars Agona, Kentucky, Newport, Saintpaul, Typhimurium, and Virchow; and, a phage λ-like prophage in serovar Schwarzengrund. The fact that *gtrABC *operons are associated with three very different temperate phage types (P22-, λ- and P2-like) suggests that these operons are evolutionarily important to the *Salmonella *temperate phages and the hosts that harbor them. The large majority of these prophage-borne *gtrABC *operons appear to be intact, but in most cases functionality has not yet been demonstrated experimentally. Nonetheless, it seems clear that *Salmonella *serotype, like *Shigella *serotype, is frequently modified by the prophages they carry. Presumably, phage carry these genes because they give their prophage host (and so the resident prophage) a selective advantage.

#### f. Control of gene expression

Gene *46 *encodes a protein that is similar to other lambdoid prophage repressors. Its C-terminal protease domain is about 80% identical to the repressors of phage λ and HK97, for example, but its DNA binding N-terminal portion is only about 50% identical to its closest known relatives in phages L, VT2-Sa and Ni12. Thus, the ε^34 ^repressor almost certainly has the same RecA-mediated inactivation mechanism in its C-terminal domain as phage λ [45], but likely has a different operator binding specificity; the ε^34 ^Cro (gene *47*) protein, which should bind the same operators as the repressor, is ≤ 42% identical to known lambdoid Cro proteins. We have identified putative P_L _and P_R _promoters (see the ε^34 ^GenBank annotation), and located just upstream of these promoters is a consensus sequence WTACRAWWTGTAT that may represent the operator sites for ε^34^.

The λ CII protein activates several promoters required for establishment of lysogeny. The ε^34 ^homologue, gp48, is weakly similar to known CII proteins in its N-terminal portion, but its C-terminal putative RNA polymerase binding domain is highly similar to other CII proteins (*e.g.*, 16 identities in the C-terminal 18 AAs with λ CII). Nonetheless, there are TTGCN_6_TTGC/T sites in reasonable positions in the predicted repressor establishment (P_RE_) and gene Q anti-sense (P_aQ_) promoter sequences that suggest the target sequence for ε^34 ^CII is the same or very similar to that of P22 and λ CII [46]. Interestingly, there are two TTGCN_6_TTGC sites upstream of *gtrA *suggesting that CII may also activate transcription of the conversion cassette in ε^34^.

Our analysis of the wildtype ε^34 ^genes *46 *(*cI*), *48 *(*cII*) and *39 *(*cIII*) and surrounding regions shows four differences from the c99 clear plaque mutant, whose genome was sequenced here. These are single amino acid changes, F46V (A32564G) in gp46, and K14E and N41S (A33236G and A33318G, respectively) in gp48 (in addition, there is a T to C change in position 32736, between genes *46 *and *47*, which does not appear to alter the rightward early promoter or its overlapping operator). The two changes in gp48 alter positions that should be in the N-terminal DNA-binding domain according to the structure of the parallel region in the phage λ CII protein [47], so the lysogeny defect(s) in c99 are almost certainly due to the above changes in gene *46 *and/or *48*. These findings confirm a previous complementation analysis which indicated that the c99 mutation was caused by a defect in two different genes [48]. In addition to the regulatory proteins mentioned above in this section, ε^34 ^encodes unambiguous homologues of the early (λ gpN) and late (λ gpQ) transcriptional antitermination proteins found in other lambdoid phages (above). These are discussed further below.

### 4. Evolution and diversity of the P22-like phages that infect *Salmonella*

#### a. P22-like prophages

**Figure 2:**
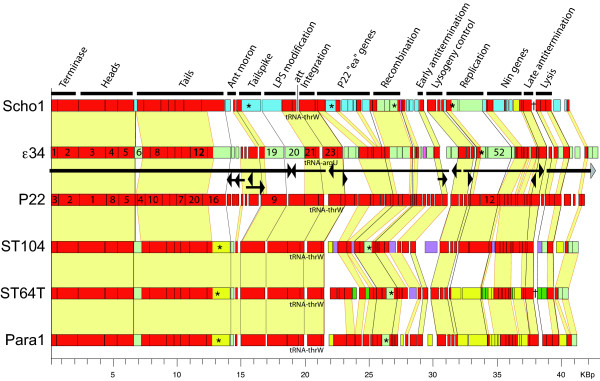
**Genomes of the P22-like phages of *Salmonella***. The genomes of four *Salmonella *temperate phages (P22, ε^34^, ST104 and ST64T) and two apparently intact *Salmonella *prophages (Scho1 and Para1; see text) are shown with the open reading frames indicated as colored rectangles. Similar rectangle colors indicate homology and these homologies are connected by yellow trapezoids between adjacent genomes; different open reading frame colors indicate apparent nonhomologies. The circular genome sequences are arbitrarily opened at the start of the small terminase gene. Above, the constant (among this type of phage) order of gene functions are indicated and think black lines between the genomes denote the apparent boundaries between these regions. Asterisks (*) mark genes where homology breaks clearly occur within genes (see text) and daggers (†) indicate the presences of tRNA genes (which read Asn GTT and Thr TGT codons in Scho1 and Asn GTT in Para1). The site of integration into the host genome is indicated at the attachment site (*att*) of each genome. Finally, the experimentally determined transcription pattern of phage P22 is indicated above the P22 genome (the gray arrowhead on the rightmost mRNA indicates that this transcript extends across the artificial break in the genome and continues at the other "end").

Several *Salmonella *phages and prophages are known to have virion assembly proteins that are *highly *related to P22 and ε^34^; phages P22, ε^34^, ST64T and ST104, and prophages in the fully sequenced genomes of *Salmonella enterica *serovar Paratyphi A strain ATCC 9150 [49] and serovar Choleraesuis strain SC-B67 [50]. These two prophages extend from genes SPA2385 through SPA2431 in Paratyphi and from SC0324 through SC0370 in Choleraesuis; we call these previously unnamed prophages "Para1" and "Scho1", respectively. Similar prophages are present in *Salmonella *serovars Arizonae (Accession No. NC_010067), Dublin (No. NZ_ABAP0100000), Hadar (NZ_ABFG01000003), and Heidelberg (NZ_ABEL01000001, NZ_ABEM01000002) that are not analyzed here because their genomes are incomplete (S. Casjens, unpublished observations). Figure 2 shows that these phages have a mosaic genome structure typical of the temperate phages (as well as most other phage types). These two prophages contain *apparently *intact orthologues of all of the genes that are known to be essential in the very well-studied phage P22. These include all of the genes required for control of phage gene expression, DNA replication, virion assembly and lysis. In theory, point mutations could have inactivated nearly any of these prophage genes, but there are many examples of fully functional genes, even in quite highly degraded prophages that are very likely much older than Scho1 and Para1 [10].

#### b. Evolution and diversity of the late operon

The morphogenetic regions of the *Salmonella *P22-like phages are much more like one another than they are like the parallel regions of P22-like phages that infect *E. coli *or *S. flexneri*. For example, the coat proteins (ε^34 ^gp5 and homologues) of the *Salmonella*-infecting members of the group listed above are all >99% identical proteins except that of Scho1 which is about 75% identical to the others. On the other hand the coat proteins of P22-like phages HK620 and CUS-3 that infect *E. coli *[51,52] and *Shigella *phage Sf6 [32] are much more distantly related, with 14–28% identity to the *Salmonella *phages. It appears that there has been little if any exchange of genetic material corresponding to the ε^34 ^gene *1–10 *region between the *Salmonella *P22-like phages and the *E. coli/Shigella *P22-like phages.

The virion assembly genes of the six *Salmonella *phage genomes are very similar (almost all exhibit >90% amino acid sequence identity), with a few exceptions as follows: (1) Very similar homologues of the phage L Dec protein (described above) are encoded by the rightmost genes of the ε^34^, ST104, ST64T and Para1 genomes in figure 2, but are not encoded by P22 and Scho1. (2) Unlike the other morphogenetic genes (noted as Terminase, Heads, Tails and Tailspike in figure 2), there are significant differences in the lengths of the homologues of ε^34 ^gene *6*. The P22 and Scho1 gene *6 *homologues have substantial internal deletions relative to the others, and their amino acid sequences have diverged substantially from the others. The role of this gene is unknown, although in P22 it is not essential in the laboratory [53]. (3) The tail and ejection proteins are more variable than the other virion assembly proteins (figure 2). The tailspike genes show an interesting relationship in which the N-terminal head-binding domains (AAs 1–115) are all quite similar; the ε^34 ^domain is the most divergent with about 75% identity to the others. The remaining C-terminal portions of the tailspikes are present in three forms (ε^34^, Scho1, and P22/ST104/ST64T/Para1) which are not recognizably similar in sequence; this receptor-recognition domain binds and cleaves the bacterial surface O-antigen polysaccharide [23,25,54]. Atomic structures of both domains of the P22 tailspike have been determined, and the C-terminal domain has an unusual β-helix structure [55,56]. The C-terminal domains of ε^34 ^and Scho1 tailspikes have high β-helix predictions [57], and the ε^34 ^tailspike is resistant to denaturing agents and proteolysis [[21,22], R. Villafane, unpublished], suggesting that they may have similar overall structures. (4) Gene *13 *of ε^34^, whose protein product is ejected with the DNA, is very similar to that of *E. coli *phage CUS-3 (not shown), so gene *13 *has clearly enjoyed exchange with phages that currently infect different host species. Thus, even in this small sampling, it is apparent that variation is greater in the genes that are thought to interact with the bacterium during the injection process. Such hyper-variation has been noticed previously in the tail fibers of long-tailed phages [58,59], but not with the short-tailed ones. Such variations are presumably the result of evolutionary sparring involving the physical interaction between the phage virions and their hosts in the first steps of infection.

**Figure 3:**
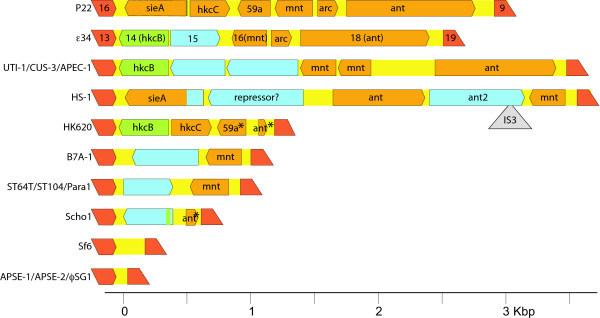
**The "ant morons" of the P22-like phages**. The genomic regions between the homologues of P22 genes *16 *and *9 *are shown for the currently known P22-like phages and prophages (the *16 *and *9 *homologues are indicated in dark orange and marked with the P22 gene name). Genes with the ant moron (see text) are shown as boxes with one end pointing the direction of transcription, and previously given gene names are indicated on the genes for phages P22 and ε^34^; gene colors and names in the other phages indicate which P22 and/or ε^34 ^they are similar to. Blue genes are each unique sequence; that is, the different blue genes are unrelated to one another. The gray triangle indicated the presence of a IS3 transposon. Asterisks (*) denote genes that appear to be damaged by truncation or frame breaking mutations relative to the P22 genes. Sources for the sequences of the phages and prophages shown in the figure, that are not given in the text, are *E. coli *HS (accession No. CP000802) and *E. coli *B7A (accession No. NZ_AAJT01000004).

Two other parts of the late operon are also of interest, the lysis genes and the "moron" immediately 5' of the tailspike gene. The lysis module of lambdoid phages consists of four genes, a holin, a lysin, and homologues of λ Rz and Rz1 proteins [60]. There are three apparently nonhomologous types of holins and three types of lysins present in the six *Salmonella *P22-like phages in figure 2, yet each of these "lysis cassettes" functions to lyse *Salmonella*. The second region, between the tailspike gene (*19*) and the rest of the late operon, is variable in length, has no function in morphogenesis or lysis, and contains different sets of genes in different phages. By these criteria this region fits the definition of a "moron", a (usually) independently expressed gene or group of genes that appears to be inserted into a phage operon when that operon is compared across various related phages [31,61]. This region, which we call here the "ant moron" because it often harbors a homologue to the P22 antirepressor gene *ant *is diagrammed in figure 3. In phage P22 this region has been studied in some depth, and it contains genes for two protein repressors (*mnt *and *arc*), an antisense RNA (*sar*) which control the expression of the antirepressor (*ant*) gene [62-67] (together these are called the *immunity I *region [68]). In addition, this region in P22 includes genes that encode a superinfection exclusion protein (*sieA*) [69] and two genes of unknown function (*hkcC, 59a*). The parallel ε^34 ^ant moron contains an apparently functional *immunity I *region (genes *16, 17 *and *18*) and two genes (*14 *and *15*) of unknown function, one of which is a homologue of gene *hkcB *which lies in a similar position in the *E. coli *phage HK620 genome [51]. The ST64T, ST104 and Para1 have ant morons that are nearly identical to one another and contain only the *mnt *repressor gene and a gene of unknown function; the parallel Scho1 region has a different gene of unknown function and what appears to be a degraded (and likely nonfunctional) Arc repressor gene. In order to further understand the variability of the ant moron, we include ten other currently known completely sequenced non-*Salmonella *P22-like phages and prophages in figure 3. These phages all have morphogenetic genes that are syntenic to P22 (but quite divergent in sequence), and they have ant moron regions that vary from its absence in Sf6 [70], APSE-1 [71], APSE-2 [72] and ϕSG1 [35] to the P22 [73]/CUS-3 [52])/UTI-1 (prophage in *E. coli *strain UT189 [74])/APEC-1 (prophage in *E. coli *strain APEC O1 [75]) types which each contain six predicted genes.

It is curious that these different morons are present at this location in these phages, but no morons are present in any other locations in their morphogenetic gene clusters. Perhaps this is the only location that can tolerate such an insertion? On the other hand, perhaps successful moron insertion is extremely rare, and since each of the ant morons has some sequence similarity to at least one of the others, it is plausible that a single ancestral moron inserted at this position in the past, and the variation that currently exists is due to more frequent insertions and deletions that modified the original insertion. From the sequence differences, it seems clear that these different morons have non-identical functions at present, and only the roles of the P22 version have been studied. Interestingly, the P22 ant moron is thought to have a (accidental?) role in controlling the level of tailspike protein produced during infection by affecting the frequency which P_Late _initiated RNA polymerase molecules transcribe through the ant moron into the tailspike gene [76]. The Mnt and Arc repressors may have a role in this process, so the ant morons of Scho1, HK620, Sf6, APSE-1, APSE-2 and ϕSG1 may lack this ability. The fact that the HK620 and Scho1 morons have what appear to be degraded *ant *genes suggests that they may at one time have had a functional antirepressor gene (and complete *immunity I *region?) in this location that is no longer needed. The apparent lack of *arc *genes in the CUS-3 and HS-1 ant type morons, in spite of the presence of putative antirepressor genes, suggests that they have a mechanism for controlling the later that differs from P22. Since all sixteen of the phages shown in figure 3 have syntenic sets of morphogenetic genes, the wide differences observed in the ant morons, ten types in sixteen genomes, indicate that this region has a much higher rate of genetic flux than the rest of the late operon.

#### c. Evolution and diversity in the early operons

The early left (early antitermination through integration regions in figure 2) and early right (replication through late antitermination regions) operons are highly mosaic, even among the *Salmonella *P22-like phages that have highly similar virion assembly genes, and there is ample evidence for past genetic exchanges with other members of the larger "lambdoid" phage group. For example, P22, ε^34 ^and Para1 have late antitermination genes that are 99% identical to phage λ gene Q protein, and putative origin recognition DNA replication proteins of ε^34 ^and Scho1 are 96% and 97% identical, respectively, to the ES18 homologue, and the parallel ST104 replication protein is 98% identical to the very different coliphage HK97 homologue (ES18 and HK97 both have long non-contractile tails and head and tail genes that are not recognizably similar to those of the P22-like phages). Clearly some of these gpQ-like and replication proteins have been exchanged independently and quite recently between the *Salmonella*-infecting P22-like phage and members of the larger lambdoid group, since they have not had time to diverge significantly from their relatives in otherwise very different phages.

#### d. DNA binding specificity of regulatory proteins

Development of the phage life cycle depends on a number of nucleotide sequence-specific nucleic acid-protein interactions. For example, in the lambdoid phages such specificity is observed in early operon repression, replication origin binding, DNA packaging, integration, and the transcriptional antiterminations that give rise to early and late transcripts [45]. For each of these, different specificities are known in different phages. These biologically important relationships are not indicated by different gene colors in figure 2 because the proteins with different specificities are nonetheless homologous to one another. Overall, the extent of diversity that is required to alter the nucleic acid binding specificity is not known (nor should it be a particular value), and of course laboratory examples are known in which single amino acid changes can alter specificity. Nonetheless, it seems reasonable to estimate that proteins with more than 90% sequence identity might be expected to have the same, or at least similar, target specificity. Table 1 shows the comparison of these six proteins in the P22-like *Salmonella *phages. Only the packaging specificity appears to be the same in all six phages. The other five proteins show between two and five different predicted specificities (*i.e*., very different sequence types). For example, each of the six repressors is very different in sequence from the others, except those of ST64T and ST104, which are identical. And the late antitermination proteins (homologues of the phage λ Q protein) of these six phages are likely to have the specificities of phages λ or Sf6, except ST104 which is an apparently "new" type. It is interesting to note that among the six ε^34 ^proteins in Table 1, three are predicted to have specificities that do not to exist in previously analyzed phages. Thus, it seems that current analyses are yet close to having a complete list of the sequence (*i.e.*, apparent specificity) types for these important proteins. This subset of these phages' genes also shows clearly that recent shuffling of these genes has occurred within the *Salmonella *P22-like phages. For example, Para1 encodes repressor and DNA replication proteins that are 100% and 97% identical to those of ST104 and ST64T, respectively, while both of these proteins are very different from each other in ST104 and ST64T. Similarly, Para1 has a phage λ (99% identity) type late antitermination protein, indicating another difference from phages ST104 and ST64T. We cannot deduce the directionality of these exchange event(s), or whether there were intermediary phages, but clearly these genes have been exchanged among these three phages so recently that one protein has not changed at all and the other only changed 3% since their last common ancestor.

**Table 1:** Predicted target specificties for ε^34 ^DNA binding proteins

	**Predicted specificity^1^**						
**DNA interaction**	**P22 gene**	**P22**	**ε^34^**	**ST64T**	**ST104**	**Para-1**	**Scho-1**

DNA packaging	*3*	P22	P22(96%)	P22(96%)	P22(96%)	P22(98%)	P22(99%)

							

Integration^2^	*int*	P22(98%) thrW	**ε^34^** argU	P22(98%) thrW	P22(98%) thrW	P22(98%) thrW	P22(98%) thrW

							

Early anti-termination	*24*	P22	**ε^34,3^**	P22(92%)	P22(98%)	P22(96%)	P22(93%)

							

Prophage repressor	*c2*	P22	**ε^34^**	L(100%)	ST104	ST104 (100%)	Scho1

							

DNA replication	*18*^5^	P22	λ (94%)^5,7^	ST64T^4^	HK97 (98%)^7^	ST64T (97%)^4^	λ (94%)^5,7^

							

Late anti-termination	*23*	λ (96%)^6^	λ (99%)	Sf6(97%)^7^	ST104	λ (99%)	Sf6(96%)

1. On each line, the table indicates (without parentheses) the experimentally-determined specificty according to the prototypical phage with that specificty. If two proteins have ≥ 92% amino acid sequence identity, the specificty is predicted to be the same, and the percent amino acid sequence identity to the prototypical protein is given in parentheses. If two unstudied proteins have <92% amino acid sequence identity their specificity is predicted to be different and one was arbitrarily chosen to be the prototype (*e.g*., ST64T DNA replication).

2. The tRNA gene into which phage integrates is shown below the *int *protein comparisons.

3. Has 72% overall amino acid sequence identity to its phage HK97 homologue, but amino acids 74–113 are very similar to the HK97 protein (see text).

4. Both have 82% amino acid sequence identity to its phage Sf6 homologue; it is not known if proteins with this level of difference could have the same target specificity.

5. N-terminal domain only (amino acids 1–162). The whole ε^34 ^and Scho1 putative replication origin recognition proteins (ε^34 ^gp50 and Scho1 gpSC0340, respectively) are 96% and 97% identical to the phage ES18 homologue [33]. Their N-terminal regions are 94% identical to phage λ gpO amino acids 17 through 148, and the N-terminal 162 amino acids have been shown to bind the origin [81].

6. Shown experimentally to have the same specificity as λ gpQ [82].

7. Phages Sf6, HK97 and λ do not infect *Salmonella*, but the indicated *Salmonella *phage protein is predicted to have the same specificity as one of these phages.

## Conclusion

We determined the complete nucleotide sequence of the *Salmonella *P22-like lambdoid phage ε^34^, and found that it has novel predicted specificities for host polysaccharide modification enzymes, virion receptor binding, integration, early transcriptional antitermination protein and prophage repressor. We used its sequence to help understand the previously unanalyzed sequences of two *Salmonella *prophages. These sequences, along with the previously known phage P22, ST64T and ST104 genome sequences, give a much clearer picture of the variation among this rather closely related group of *Salmonella *phages. In spite of this very close relationship, genome mosaicism is found to be prevalent in the early regions of their genomes.

## Methods

### Purification of phage and isolation of DNA

The original phage strain, ε^34^, a generous gift from Dr. Andrew Wright (to RV) and Dr. Horst Schmieger (to SC), was subjected to hydroxylamine mutagenesis to produce the clear plaque and highly lytic variant, ε^34 ^c99 [48,77]. The mutant and wildtype DNAs were prepared and purified as described [21,78] or by using QIAGEN Lambda DNA Purification Kit (QIAGEN, Valencia CA).

### DNA sequencing

The DNA was sequenced commercially by Fidelity System Inc. (Gaithersburg MD) with the final sequence opened immediately upstream of the small terminase subunit gene to be in conformity with the presentation of other similar phages. The completely annotated sequence for this phage can be obtained from GenBank under Accession No. EU570103. This sequence agrees with previously determined sequences of the ε^34 ^tailspike gene [25] and scaffolding protein gene (P. Weigele and S. Casjens, unpublished).

### Annotation

Protein-encoding genes were identified using Kodon (Applied Maths, Austin, TX) with the proteins being screened for homologues using BLASTP and PSI-BLAST [28] against the nonredundant protein database at NCBI. Terminators, promoters and operator sequences were defined on the basis of their position and sequence relatedness to similar sites in *Salmonella *phage P22. The Betawrap [57], Softberry , tRNAscan-SE [79], [44] web sites were used for beta-helix structure, phage promoter, tRNA gene and transmembrane helix identification, respectively.

## Abbreviations

gpX: gene product of gene X

## Authors' contributions

RV, AK and SC jointly conceived of the project, EG, MZ and RV carried out preliminary and finalization sequencing work, and AK completed the nucleotide sequence, and RV, SC and AK cooperated to perform the sequence analysis and draft the manuscript. All authors have read and approved the final manuscript.

## Supplementary Material

Additional file 1 **Contains Figure S1 showing gene map of phage ε^34 ^and a Table S1 that lists the putative deduced function of each ε^34 ^gene and some of its homologues in the current sequence database**. Click here for file
