# Supplementary material for: Genomic analysis of bacteriophage ε34 of Salmonella enterica serovar Anatum (15+)
Source: BMC Microbiol. 2008 Dec 17;8:227. doi: 10.1186/1471-2180-8-227 (PMC2629481; doi:10.1186/1471-2180-8-227)
Supplement: Additional file 1 — Contains Figure S1 showing gene map of phage ε34 and a Table S1 that lists the putative deduced function of each ε34 gene and some of its homologues in the current sequence database. [file 1471-2180-8-227-S1.pdf]

SUPPLEMETARY MATERIAL

to

Genomic analysis of bacteriophage e^{34} of *Salmonella enterica* serovar Anatum (15+)

by

Robert Villafane, Milka Zayas, Eddie B. Gilcrease,

Andrew M. Kropinski and Sherwood R. Casjens

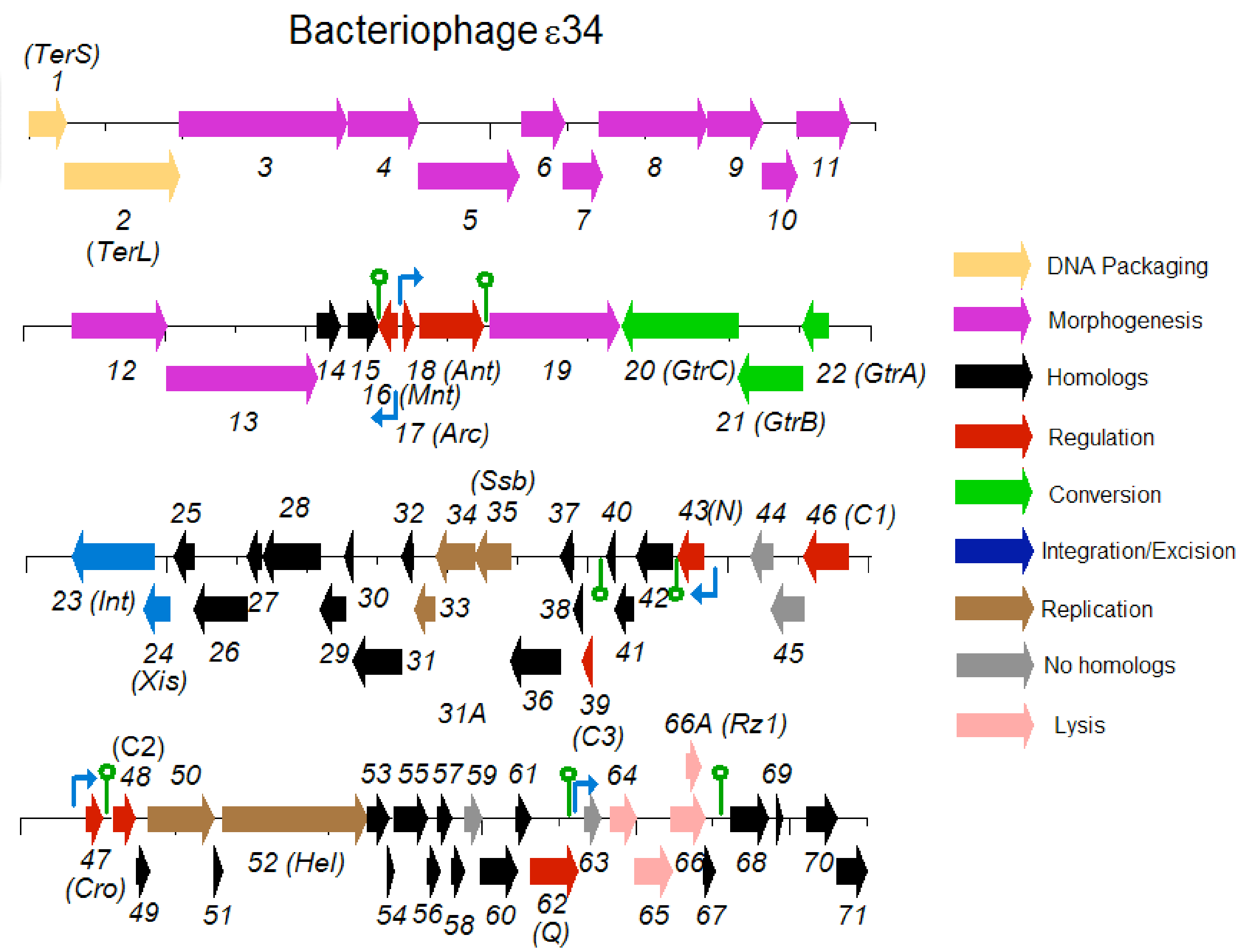

Supplementary Figure S1. The bacteriophage e^{34} genome. The genome sequence of ε^{34} is opened arbitrarily at the start of the small terminase (terS) gene, and predicted genes are shown as filled arrows. Predicted promoters and transcription terminators are indicated by small blue arrows and green circles, respectively.

Supplementary Table S1

Phage ε^{34} genes and their putative functions

CDS	Position and orientation	Amino acid Residues	Putative Function	Phage Homologs	Identity
1	1-489>	162	Small terminase subunit; recognition of DNA for packaging	Gp3 (**Terminase small subunit**) of *Salmonella* Typhimurium phages ST64T (NP_720325), ST104 (YP_006404), PS3 (CAA09703), & PS119 (CAA09712)	160/162
2	467-1966>	499	Terminase large subunit; DNA packaging motor ATPase;	Gp2 (**Terminase large subunit**) of phages ST64T (NP_720326) Gp2 L (AAX21426) & P22 (YP_063734)	495/499 [517] 496/499
3	1966-4143>	725	Portal protein (part of DNA packaging motor and portal through which DNA enters capsid)	Gp1 (**Portal protein**) phages ST104 (YP_006406); ST64T (NP_720327); P22 (YP_063735)	716/725 715/725 711/725
4	4157-5068>	228	Scaffolding protein (catalytic in procapsid assembly)	Gp8 (**Scaffolding protein**) phages P22 (YP_063736); ST64T (NP_720328); ST104 (YP_006407)	301/303 299/303 298/303
5	5068-6360>	430	Coat protein (icosahedral head shell)	Gp5 (**Coat protein**) phages ST64T (NP_720329) & ST104 (BAD15215) P22 (NP_059630)	427/430 423/430

6	6401-6961>	186	Unknown function; related to apparently partially deleted Orf109 of phage P22	ORF54 phage ST104 (YP_006409)<br>Orf-186 ST64T (NP_720330)	186/186<br><br>177/186
7	6945-7445>	166	Tail accessory protein	Gp4 (**Head completion protein**) phages ST104 (YP_006410)<br>P22 (NP_059632)<br>ST64T (NP_720331)	162/166<br><br>161/166<br>160/166
8	7405-8823>	472	Tail accessory protein	Gp10 (**Head completion protein**) phages ST64T (NP_720332)<br>ST104 (YP_006412)<br>P22 (NP_059633)	470/472<br><br>463/472<br>452/472
9	8827-9528>	233	Tail accessory protein	Gp26 (**Head completion protein**) phages ST104 (YP_006413)<br>P22 (YP_063715)	221/233<br><br>188/233
10	9528-9983>	151	Unknown (required for virion assembly at high temperature)	Gp14 phages ST64T (NP_720334) & ST104 (YP_006414)<br>**Virion assembly protein** HK620 (NP_112084)	148/151<br><br>146/151
11	9986-10675>	229	Ejection protein (ejected with DNA during injection; required for successful DNA injection)	Gp7 (**DNA transfer protein**) phage ST64T (NP_720335)	221/229
12	10685-12034>	449	Ejection protein	Gp20 (**DNA injection protein**) phage ST64T (NP_720336)	415/443
13	12031-14157>	708	Ejection protein	**DNA transfer protein** phage HK620 (NP_112087)	507/691 [722]
14	14158-14481<	107	Unknown function	**HkcB** protein phage HK620 (NP_112088)	53/100

15	14600-15019>	139	Unknown function	CHP *Actinobacillus succinogenes* (YP_001345330)	71/132
16	15036-15287<	83	Transcriptional repressor Mnt	Transcriptional repressor **Mnt** phages P22 (NP_059641)<br>Mnt ST64T (NP_720338) & ST104 (YP_006419)	80/83<br><br>50/81
17	15378-15539>	53	Transcriptional repressor Arc	Transcriptional repressor **Arc** phage P22 (NP_059642)	51/53
18	15608-16510>	300	Anti-repressor protein	Antirepressor **Ant** phage P22 (NP_059643)	259/300
19	16611-18431>	606	Tailspike protein	Gp14 (tailspike protein) phage Sf6 (NP_958190)<br>Gp9 (**tailspike protein**) HK620 (NP_112090)	121/197 [623] Nterm<br>109/158 [710] Nterm
20	18467-20122<	551	Probable O-antigen modification	**GtrC** *Salmonella* Paratyphi HP SPAB_03004 (YP_001589201)	215/558
21	20122-21039<	305	Glycosyl transferase	**GtrB** phages ST64T (NP_720276)<br>ST104 (YP_006358)<br>P22 (NP_059582)	258/304 [325]<br>258/304<br>257/304
22	21036-21398<	120	Bactoprenol-linked glucose translocase (flipase)	Bactoprenol-linked glucose translocase phages SfX (GTRA_BPSFX)<br>**GtrA** SfII (GTRA_BPSF2)	70/120<br><br>66/120
23	21661-22824<	387	Tyrosine integrase	**Integrase** phages SfV (NP_599058)<br>ST104 (YP_006360) & ST64T (NP_720278)	271/386<br><br>270/383

24	22680-23051<	123	Excisionase	**Excision protein Xis** phages P22 (NP_059585) & ST64T (NP_720279)<br>Xis ST104 (YP_006361)	91/116<br><br>88/116
25	23114-23386<	90	Unknown function	HP Gp48 phage ε15 (NP_848256)	88/90
26	23386-24141<	251	Unknown function	HP Gp47 phage ε15 (NP_848255)	251/251
27	24145-24363<	72	Unknown function	HP Gp46 phages ε 15 (NP_848254)<br>HP Gp39 ES18 (YP_224177)	71/72<br><br>65/72
28	24365-25198<	277	Unknown function	**EaD** protein phage ε 15 (NP_848253)<br>EaD P22 (YP_063721)	271/277<br><br>188/197
29	25195-25554<	119	Unknown function	**EaF** phages P22 (YP_063722)	119/119
30	25529-25666<	45	Unknown function	HP orf45 phage P22 (YP_063723)	45/45
31A	25663-26229<	188	Homing endonuclease	Putative **HNH endonuclease** phage φAT3 (YP_025078)	57/171 [222]
31	25663-26355<	230	N-term 44 AAs 93% identical to ST64T EaE	**EaE** phage ST64T (NP_720287)<br><br>Putative HNH endonuclease phage φAT3 (YP_025078)	41/46 [125] Nterm<br>57/171 [222] Cterm
32	26352-26522<	56	Unknown function	ORF10 phage ST104 (YP_006366)<br>Orf56 ST64T (NP_720288)	53/56<br><br>53/56

33	26533-26826<	97	Anti-RecBCD protein (Abc2)	Anti-RecBCD protein 2 phages P22 (NP_059594)<br>**Abc2** ST64T (NP_720289)<br>Gp39 HK97 (NP_037724) & Abc2 HK022 (NP_037689)	93/97<br><br>92/97<br>88/97
34	26842-27390<	182	Putative exodeoxyribonuclease VIII	Endodeoxyribonuclease phages ST64B (NP_700403)<br>Exonuclease PY54 (NP_892096)	 107/181<br>105/183
35	27399-27905<	168	Bacterial type single-strand binding protein	**SSB** phage CP-1639 (CAC83134)<br>Gp48 ES18 (YP_224186)<br>Gp27 Sf6 (NP_958203)	119/168<br><br>108/156<br>106/157
36	27906-28613<	235	Homologous recombination; C-terminal domain (62 AA) identical to P22 Erf	Orf-235 phage ST64T (NP_720291)<br>Gp49 ES18 (YP_224187)<br>ORF13 ST104 (YP_006369)	226/235<br><br>222/235<br>219/235
37	28622-28810<	62	Unknown function	Orf-66 phage ST64T (NP_720292)<br>Gp50 ES18 (YP_224188)	62/62<br><br>61/62
38	28807-28920<	37	Inhibition of cell division; phage lamda Kil protein	**Kil protein** phage ST64T (NP_720293) & ES18 (YP_224189)	37/37<br>37/37
39	28913-29059<	48	Establishment of lysogeny; phage lambda CIII protein inhibitior of host HflB protease	**C3** phage ST64T (NP_720294)<br>C3 ST104 (YP_006371)<br>C3 P22 (NP_059599)	45/48 [55]<br>41/45 [52]<br>34/45 [52]
40	29264-29389<	41	Unknown function	*Enterobacter sakazakii* HP ESA_03093 (YP_001439158)	 34/41
41	29389-29667<	92	Unknown function	ORF18 phage ST104 (YP_006373)	73/95

42	29701-30210<	169	Unknown function; 12 imperfect repeats of pentapeptide ADLRD	Orf-232 phage ST64T (NP_720296)<br>ORF19 phage ST104 (YP_006374)	154/183 [232]<br>156/214
43	30289-30651<	120	Transcriptional anti-termination; phage lambda N protein	Putative **N protein** phage φ4795 (NP_851956)<br>N protein H-19B (AAD04643)	92/127 [149]<br>92/127
44	31323-31634<	103	Unknown function	-	
45	31621-32091<	156	Unknown function	-	
46	32091-32729<	207	Transcriptional repressor, phage lambda CI protein	**CI protein** Stx1 converting phage I (NP_859200) & II (NP_859200)	147/208 [231]
47	32854-33063>	69	Transcriptional repressor, phage lambda Cro protein	C2 phages VT2-Sa (NP_050523)<br>Stx1 phage (NP_859202),<br>Stx2 phage (NP_859368),<br>& **Cro** ST64T (NP_720300)	29/67<br>29/67<br>29/67<br>29/67
48	33197-33487>	96	Establishment of lysogeny; phage lambda CII protein transcriptional activator	**CII protein** phage 21 (CAB39983)<br>cII VT2-Sa, StxI (NP_859203) & StxII (NP_859369)	29/90<br><br>32/98<br>32/98
49	33508-33669>	53	Unknown function	Gp58 phage ES18 (YP_224196)<br>Gp53 HK97 (NP_037738),<br>gene 42 Sf6 (NP_958217),<br>& ORF27 ST104 (YP_006382)	51/53<br>52/53<br>52/53<br>52/53
50	33656-34504>	282	Initiation of DNA replication; N-terminal 139 AAs are 90% identical to DNA-binding portion of phage lambda gpO	Gp59 (**replication protein O**) phage ES18 (YP_224197)	275/282
51	34508-34615>	35	Unknown function	Gp60 phage ES18 (YP_224198)	24/33
52	34615-36495>	626	Probable function in DNA replication	Gp61 phage ES18 (YP_224199)	611/626

53	36496-36774>	92	Unknown function	Gp62 phage ES18 (YP_224200)	80/92
54	36771-36851>	26	Unknown function; Phage lambda NinA	Gp45 phage Sf6 (NP_958220)<br>Gp63 phage ES18 (YP_224201)<br>**NinA** NinA 21 (CAB39987)	23/25<br>24/26<br>23/26
55	26848-37285>	145	Homologous recombination; phage lambda NinB	**NinB** phage ST104 (YP_006386)<br>NinB P22 (NP_059612) & Gp64 ES18 (YP_224202)	144/145<br>143/145
56	37282-37455>	57	Unknown function; Phage lambda NinD	**NinD** phage P22 (YP_063726)<br>NinD ST104 (YP_006387) & ES18 (YP_224203)	57/57<br>56/57
57	37422-37604>	60	Unknown function; Phage lambda NinE	**NinE** phage ST64T (NP_720310)<br>NinE ST104 (YP_006388)<br>NinE P22 (NP_059614)	58/58<br>57/58<br>56/58
58	37601-37771>	56	Unknown function; Phage lambda NinF	HP phage HK620 (NP_112062)<br>**NinF** HK97 (NP_037746),<br>86 (YP_794123)	54/56<br>53/56<br>53/56
59	37764-38000>	78	Unknown function	-	
60	37981-38451>	156	Unknown function	*Salmonella* Arizonae HP SARI_02660 (YP_001571659)	148/156
61	38439-38621>	60	Unknown function; Phage P22 NinZ	**NinZ** phage ST64T (NP_720317)<br>NinZ P22 (YP_063729) & ST104 (YP_006394)	55/58<br>53/58
62	38618-39241>	207	Transcriptional anti-termination of late operon; phage lambda Q protein	**Antitermination protein Q** phage lambda (NP_040642)<br>Q phage HK97 (NP_037751), HK022 (NP_037694) & HK620 (NP_112067)	205/207<br><br>204/207
63	39331-39540>	69	Unknown function	-	

64	39676-40002>	108	Phage holin; phage lambda lysis protein S	Gp60 protein phage Sf6 (NP_958234)<br>**Holin** (gpS protein) lambda (NP_040644),<br>HK620 (NP_112068)	105/106<br>104/106<br>104/106
65	39986-40462>	158	Endolysin (transglycosidase); phage lambda gene R protein;	Lysin phage HK97 (NP_037753)<br>& HK620 (NP_112069)'<br>**Lysin** HK022 (NP_037696)	157/158<br>157/158<br>156/158
66	40459-40896>	145	Lysis in presence of divalent cations	Gp63 protein phage Sf6 (NP_958237)<br>**Rz lysis protein** HK97 (NP_037754)<br>Gp15 PS34 (CAA09707)	141/145<br>139/145<br>135/145
66A	40658-40843	61	Lysis in presence of divalent cations	Gp64 phage Sf6 (NP_958238)<br>**Rz1 lysis protein** P22 (YP_063732)	59/61<br>57/61
67	40884-41036>	50	Unknown function	*Escherichia coli* HP UTI89_C2658 (YP_541655),<br>Sf6 gene 65 (NP_958239)	49/50<br>49/50
68	41241-41726>	161	Unknown function	HP phage HK022 (NP_597906)<br>& HK620 (NP_112072)	18/55 [85]<br>18/55 [85]
69	41974-42216>	80	Unknown function	HP phages HK620 (NP_112073)<br>& Sf6 (NP_958241)<br>Orf80 P22 (NP_059625)	80/80<br>80/80<br>79/80
70	42220-42609>	129	Unknown function	Orf-129 phage ST64T (NP_720323) &<br>ORF47 ST104 (YP_006402)	124/129<br>124/129

71	42609-43013>	134	Decoration protein; capsid stability	Orf-134 phage ST64T (NP_720324),<br>ORF48 ST104 (YP_006403),<br>Phage L Dec protein (AAX21424)	130/134<br><br>130/134<br>130/134

> = CDS in plus strand
< = CDS in minus strand
Nterm = homology only at the N terminus of the protein
HP = hypothetical protein
Cterm = carboxy terminus is homologous
CHP = conserved hypothetical protein (homologs in bacteria)
[ ] = length of homolog in amino acid residues
